# Does working capital management improve financial performance in China’s agri-food sector during COVID-19? A comparison with the 2008 financial crisis

**DOI:** 10.1371/journal.pone.0300217

**Published:** 2024-04-03

**Authors:** Lujing Liu, Xiaoning Zhou, Jian Xu

**Affiliations:** School of Economics and Management, Qingdao Agricultural University, Qingdao, China; University of Cagliari: Universita degli Studi Di Cagliari, ITALY

## Abstract

The objective of this study is to explore the impact of working capital management on firms’ financial performance in China’s agri-food sector from 2006 to 2021. In addition, we analyze whether this impact is the same during the 2008 financial crisis and the 2020 COVID-19 crisis. Working capital management is measured by working capital investment policy (measured by current assets to total assets ratio), working capital financing policy (measured by current liabilities to total assets ratio), cash conversion cycle, and net working capital ratio. The results reveal that current assets to total assets ratio and net working capital ratio positively influence financial performance measured through return on assets (ROA), while current liabilities to total assets ratio and cash conversion cycle negatively influence ROA. We also find that the relationship between working capital management and financial performance is more affected during COVID-19 than in the 2008 financial crisis. The findings might provide important implications for company managers to make optimal working capital management practices, depending on the economic environment.

## 1. Introduction

Working capital (WC), defined as the difference between current assets and current liabilities, is a measure of a company’s operating efficiency and short-term financial position [1–4]. Positive WC means that there are more current assets than current liabilities within a firm, and negative WC suggests that current assets are not enough to pay for all current liabilities [5]. WC policies adopted by companies play a vital role in corporate finance, which can directly affect corporate growth and performance [6, 7].

Working capital management (WCM) is a challenge for financial managers whose decision can affect financial position of firms [8–10]. It is related to the management of cash, accounts receivable, and inventory [2]. Previous research has stressed the importance of efficient WCM, especially during the global economic recession [11–15]. In late 2019, the coronavirus disease 2019 (COVID-19) broke out in Wuhan, China and then quickly spread around the world [16], causing the disruption of supply chain, the decline of business sales and performance, and the downturn of the global economy [17, 18]. Businesses witnessed significant deterioration in the performance of their WCs by the delay of payments and the increase in accounts receivable [19]. It was reported by PricewaterhouseCoopers (PwC), a consultant company, that net WC days touched 5 years high in 2020 [20]. Therefore, analyzing how companies can efficiently carry out WCM in the era of COVID-19 is of great importance.

Most researchers have looked at the financial effect of COVID-19 from a macro perspective [21, 22], and the impact of COVID-19 on corporate WCM is still a heated discussed topic with contradictory results [5]. In the existing literature, Zimon and Tarighi [19] pointed out that COVID-19 does not change WCM of Polish small and medium-sized enterprises. Yousaf [23] concluded that the efficiency of WCM is severely affected by the COVID-19 crisis. Simon et al. [24] reported that traditional method of WCM is no longer appropriate in a crisis. In addition, He et al. [25] pointed out that the impact of COVID-19 is different from the 2008 financial crisis. Ahmad et al. [5] argued that the WCM-firm performance relationship is more affected during COVID-19 than the 2008 financial crisis. Few studies in the relevant WCM literature compare COVID-19 with the 2008 financial crisis. Therefore, understanding the difference in WCM during both COVID-19 and the financial crisis of 2008 will aid managers in making effective WCM strategies to deal with profit deterioration.

The need for efficient WCM in both crisis and normal periods has been demonstrated in previous studies in developing countries [26, 27]. Since China is among the few countries that had successfully control COVID-19, we aim to fill this gap by examining the WC-financial performance relationship in China’s agri-food sector during both COVID-19 and the financial crisis of 2008. Specifically, we examine the impact of WC policies and WCM on financial performance measured through return on assets (ROA). WC policies include investment policy and financing policy, and the components of WCM consist of cash conversion cycle (CCC) and net working capital ratio (NWC). In robustness check, return on equity (ROE) is an alternative for financial performance.

The current study could make contributions to the WC literature in three ways. First, this is among the first studies to explore the impact of WC (including WC policies and WCM) on financial performance of agri-food companies during COVID-19 in the case of China, which extends the current literature on WC practices in crisis. In addition, most studies on WCM focus on manufacturing sector [3, 28–30], and very little has been done in agri-food sector. Second, few studies have compared the difference in WC practices during COVID-19 and the 2008 financial crisis, and we attempt to fill this gap. Finally, the findings will aid agri-food companies to better manage WC in crisis. This study can also contribute to help the governmental institutions implement favorable policies to stimulate WC performance in face of crisis.

This study is structured as follows. Section 2 presents the literature review and develops the testable hypotheses. Section 3 provides the research methodology. In Section 4, the empirical results are presented and discussed. Finally, the conclusion is provided in Section 5.

## 2. Literature review and hypotheses development

### 2.1 WC policies and firms’ financial performance

Effective WCM can optimize operating costs and encourage firms’ financial performance [19]. In the WC literature, there are two types of policies, namely WC investment policy and WC financing policy [5]. The former relates to current asset levels measured by the ratio of current assets to total assets, which incorporates aggressive investment policy and conservative investment policy [31]. The objective of an aggressive investment policy is to achieve high returns. Under this policy, corporate management is more likely to invest in risky projects to achieve higher returns. Firms with aggressive WC investment policy tend to have low investments in current assets, which can lead to WC inadequacy. However, conservative investment policy enables companies to make high investments in current assets, which can cause a high cost of liquidity. Corporate leaders are risk averse under conservative investment policy, and ensuring the safe and normal operation of the business becomes their most important task.

WC financing policy relates to the utilization of current liabilities to finance current assets, which is measured by the ratio of current liabilities to total assets [5]. It can be also divided into aggressive financing policy and conservative financing policy. Aggressive financing policies indirectly maximize returns by reducing the cost of financing. Under aggressive financing policy, companies tend to finance current assets with short-term debt. This policy is characterized by the fact that corporate current liabilities are matched with current assets and part of non-current assets. However, companies finance current assets with long-term debt under conservative financing policy [32]. The proportion of non-current assets is higher, which increases financing costs and reduces business returns, but due to the long repayment period of non-current liabilities, the debt repayment risk is smaller. For financial managers, they should reasonably consider WC policies in order to ensure sustainable firm performance.

The prior literature has not reached a consensus on WC policies and firm performance [19]. Tahir and Anuar [33] concluded that WC investment policy has a positive association with profitability while WC financing policy has a negative impact. A study carried out by Rasyid [34] in Indonesia showed a positive relationship between WC investment policy and firm performance and a negative relationship between WC financing policy and firm performance. For Thai firms, Pestonji and Wichitsathian [35] found the same results. Farhan et al. [36] argued that all firms in India follow conservative investment and financing policy. They also found that conservative investment policy improves ROA, while conservative financing policy reduces ROA. Basyith et al. [37] claimed that WC investment policy positively affects ROA, while WC financing policy negatively influences ROA and gross profit margin. However, Nabi et al. [32], taking cement and sugar companies as the sample, concluded that companies with aggressive investment and financing policies yield low profits. Al-Mawsheki [6] pointed out that WC investment policy has a negative impact on financial performance while WC financing policy has a positive impact in Malaysian manufacturing sector. Therefore, the following hypotheses are proposed:

**Hypothesis 1 (H1).** *WC investment policy positively affects firms’ financial performance in China’s agri-food sector*.
**Hypothesis 2 (H2).** *WC financing policy negatively affects firms’ financial performance in China’s agri-food sector*.

### 2.2 WCM and firms’ financial performance

Contingency theory suggests that the dynamically changing environment is affected by many factors, and the internal and external factors in any organization are different. Therefore, there is no universally applicable management style. In WCM, the daily economic activities of companies are influenced by both internal and external factors (e.g. policies, consumer preferences, and economic environment), and a uniform management style is not scientific [38]. Companies need to choose different ways of WCM according to the actual situation in order to promote the optimization and improvement of WCM.

The early theory of WCM divides a company’s WCM into the management of current assets and current liabilities and analyzes each component of current assets and current liabilities including cash, inventories, accounts receivable, short-term borrowings, and accounts payable. Some indicators such as CCC and NWC can be used to measure WCM [5, 13, 39–40]. CCC measures the average time a firm takes from purchasing materials to collecting sales [41]. The shorter CCC, the better firm performance. NWC measures whether current assets are sufficient to cover current liabilities when converted into cash. Previous literature has produced mixed results regarding how WCM affects firms’ financial performance.

Regarding CCC, Chang [42] observed that it has a negative influence on firm profitability. As Dalci and Ozyapici [43] argued, increasing the length of CCC can decrease hospital profitability. Pham et al. [44] and Nguyen et al. [45] reported that CCC negatively affects firm performance in Vietnam. Mandipa et al. [46] concluded that CCC and financial performance are negatively related in South African retail sector. In another emerging economy (i.e. India), Farhan et al. [47] reported that CCC is negatively related to ROA, net profit margin, and Tobin’s Q. Özkaya and Yaşar [10] also found a negative relationship between them. However, Laghari and Chengang [48] documented a positive impact of CCC on Chinese companies’ performance. For Ethiopian exporting firms, Wassie [49] observed a positive CCC-ROA relationship. Panigrahi et al. [29] suggested that CCC has a positive impact on ROA while days in WC have a negative impact. In the study of Korent and Orsag [50], higher profitability of firms in Croatia is related to CCC. In addition, Liu and Xu [2] found no significant relationship between CCC and the profitability of Chinese agricultural companies.

As for NWC, Senan et al. [51] found that it increases the value of Indian firms. In a study of 28 companies in Tehran Stock Exchange, Javanmardi et al. [52] found that net WC is an important ratio in the ranking of companies. Kam et al. [53] pointed out that the ratio of net WC to sales is positively related to ROA in the negative WC group while it is negatively related in the positive WC group. For manufacturing firms during 2016–2019, Yanti [54] argued that net WC is positively related to cash holdings, thus improving firm performance. Michalski [55] concluded that low levels of WC lead some firms to negative changes in sales, thus reducing profits. However, Tahir and Anuar [33] claimed that net WC level has a negative impact on ROA. Dimitropoulos and Asteriou [56] suggested that NWC and the ratio of net profit to sales have a negative impact on stock returns. A study carried out in Poland by Anton and Afloarei Nucu [57] revealed an inverted U-shaped relationship between NWC and ROA. Similarly, the same results were found by Afrifa [58]. Therefore, the third and fourth hypotheses are proposed as follows:

**Hypothesis 3 (H3).** *CCC negatively positively affects firms’ financial performance in China’s agri-food sector*.
**Hypothesis 4 (H4).** *NWC positively affects firms’ financial performance in China’s agri-food sector*.

### 2.3 WC and firms’ financial performance during crisis

There is evidence that economic conditions can change WC strategies [24, 59], and little has been done on the role of WC policies during times of crisis [5]. A study by Rozari et al. [60] revealed that the positive relationship between WC investment policy and Indonesian firm performance becomes weaker in the financial crisis of 2008. Ahmad et al. [5] concluded that the COVID-19 crisis has a more devastating influence on the WC-firm performance relationship. Companies need urgently to apply conservative WC and withdraw accounts receivable during COVID-19. It was reported that the COVID-19 impacts world more profoundly than the 2008 financial crisis. Therefore, the following hypotheses are developed as follows:

**Hypothesis 5 (H5).** *The positive relationship between WC investment policy and financial performance is stronger during COVID-19 than that in the 2008 financial crisis*.
**Hypothesis 6 (H6).** *The negative relationship between WC financing policy and financial performance is stronger during COVID-19 than that in the 2008 financial crisis*.

The empirical results on the impact of crisis on WCM are inconsistent. According to Enqvist et al. [61], WCM is more pronounced during economic downturns. In a study, it is found that WCM becomes more important during 2008 crisis compared to pre-crisis period [62]. Akbar et al. [12] proved that CCC is positively associated with ROE during global crisis. Using a case study, Wadesango et al. [63] argued that the sample experienced poor WCM practices because of the COVID-19, thus reducing firm profitability. Demiraj et al. [30] pointed out that CCC has a negative influence on ROA in the automotive sector before and during COVID-19. Safitri et al. [64] reported a non-significant relationship between CCC and ROA before and during the COVID-19 pandemic. Corporate managers put more emphasis on maintaining sufficient levels of WC in order to survive in the COVID-19 era. Therefore, the following hypotheses are developed as follows:

**Hypothesis 7 (H7).** *The negative relationship between CCC and financial performance is stronger during COVID-19 than that in the 2008 financial crisis*.
**Hypothesis 8 (H8).** *The positive relationship between NWC and financial performance is stronger during COVID-19 than that in the 2008 financial crisis*.

## 3. Methodology

### 3.1 Sample

Our sample includes agri-food companies listed on the Shanghai and Shenzhen stock exchanges during the 2006–2021 period. The 2008–2009 period is considered as the 2008 financial crisis and the period between 2020 and 2021 is considered as the COVID-19 period. After screening our sample, companies with incomplete information, companies listed after 2006, companies with the change in main business during this period, and special treatment (ST) companies are removed. Finally, 8 companies with 128 firm-year observations are left for further analysis. These 8 representative companies are the leading companies in China’s agri-food sector, and they have been operating for more than 20 years. Secondary data are retrieved from the China Stock Market Accounting Research (CSMAR) database.

### 3.2 Variables

1. Dependent variables. There is much literature that examined the effect of WC on firms’ financial performance by employing accounting-based measures such as ROA, ROE, and net profit margin [2, 13, 33, 35, 36, 46]. ROA and ROE are the most widely used proxies of financial performance [18, 65, 66]. In this study, ROA is used to measure financial performance of agri-food companies, and ROE (measured by dividing net income by total equity) is used in the robustness check.
2. Independent variables. To measure WC policies, current assets to total assets ratio (CATA) is used for WC investment policies, and current liabilities to total assets ratio (CLTA) is used for WC financing policies, consistent with previous literature [5, 6, 33, 67]. Following Liu and Xu [2], Ahmad et al. [5], and Tarkom [68], CCC and NWC are applied to measure WCM.
3. Control variables. With reference to Liu and Xu [2], Ahmad et al. [5], Al-Mawsheki [6], Mardones [9], and Korent and Orsag [50], firm size (SIZE) and firm leverage (LEV) as firm-specific control variables and gross domestic product growth rate (GDP) as country-specific control variable are included. In addition, we aim to analyze whether the impact of COVID-19 on the relationship between WC and financial relationship is stronger than the 2008 financial crisis, two dummy variables (CRISIS and COVID) are introduced. CRISIS takes a value of 1 if the year is 2008 and 2009 or 0 otherwise, and COVID takes a value of 1 if the year is 2020 and 2021 or 0 otherwise.

Table 1 shows the definition of all variables.

**Table 1:** Variable definition.

Variable	Symbol	Measurement
Return on assets	ROA	Net income/total assets
Current assets to total assets ratio	CATA	Current assets/total assets
Current liabilities to total assets ratio	CLTA	Current liabilities/total assets
Cash conversion cycle	CCC	Inventory conversion cycle + receivable collection cycle–payable deferral cycle
Net working capital	NWC	(Current assets–current liabilities)/total assets
2008 financial crisis	CRISIS	Dummy variable that takes 1 if the year is 2008 and 2009, 0 otherwise
COVID-19 crisis	COVID	Dummy variable that takes 1 if the year is 2020 and 2021, 0 otherwise
Firm size	SIZE	Natural logarithm of total assets
Firm leverage	LEV	Total liabilities/total equity
Gross domestic product growth rate	GDP	Growth rate of gross domestic product

### 3.3 Models

Models (1)–(3) examine the impact of WC on firms’ financial performance in China’s agri-food sector.


$$ROA_{i,t}={ß}_{0}+{ß}_{1}CATA_{i,t}+{ß}_{2}CLTA_{i,t}+{ß}_{3}SIZE_{i,t}+{ß}_{4}LEV_{i,t}+{ß}_{5}GDP_{i,t}+\varepsilon_{i,t}$$ (1)

$$ROA_{i,t}={ß}_{0}+{ß}_{1}CCC_{i,t}+{ß}_{2}SIZE_{i,t}+{ß}_{3}LEV_{i,t}+{ß}_{4}GDP_{i,t}+\varepsilon_{i,t}$$ (2)

$$ROA_{i,t}={ß}_{0}+{ß}_{1}NWC_{i,t}+{ß}_{2}SIZE_{i,t}+{ß}_{3}LEV_{i,t}+{ß}_{4}GDP_{i,t}+\varepsilon_{i,t}$$ (3)

Models (4)–(9) are used to examine the impact of these two crises on the WC-financial performance relationship.

$$ROA_{i,t}={ß}_{0}+{ß}_{1}CRISIS_{i,t}+{ß}_{2}CRSIS_{i,t}\times CATA_{i,t}+{ß}_{3}CRSIS_{i,t}\times CLTA_{i,t}+{ß}_{4}SIZE_{i,t}+{ß}_{5}LEV_{i,t}+{ß}_{6}GDP_{i,t}+\varepsilon_{i,t}$$ (4)

$$ROA_{i,t}={ß}_{0}+{ß}_{1}CRISIS_{i,t}+{ß}_{2}CRSIS_{i,t}\times CCC_{i,t}+{ß}_{3}SIZE_{i,t}+{ß}_{4}LEV_{i,t}+{ß}_{5}GDP_{i,t}+\varepsilon_{i,t}$$ (5)

$$ROA_{i,t}={ß}_{0}+{ß}_{1}CRISIS_{i,t}+{ß}_{2}CRSIS_{i,t}\times NWC_{i,t}+{ß}_{3}SIZE_{i,t}+{ß}_{4}LEV_{i,t}+{ß}_{5}GDP_{i,t}+\varepsilon_{i,t}$$ (6)

$$ROA_{i,t}={ß}_{0}+{ß}_{1}COVID_{i,t}+{ß}_{2}COVID_{i,t}\times CATA_{i,t}+{ß}_{3}COVID_{i,t}\times CLTA_{i,t}+{ß}_{4}SIZE_{i,t}+{ß}_{5}LEV_{i,t}+{ß}_{6}GDP_{i,t}+\varepsilon_{i,t}$$ (7)

$$ROA_{i,t}={ß}_{0}+{ß}_{1}COVID_{i,t}+{ß}_{2}COVID_{i,t}\times CCC_{i,t}+{ß}_{3}SIZE_{i,t}+{ß}_{4}LEV_{i,t}+{ß}_{5}GDP_{i,t}+\varepsilon_{i,t}$$ (8)

$$ROA_{i,t}={ß}_{0}+{ß}_{1}COVID_{i,t}+{ß}_{2}COVID_{i,t}\times NWC_{i,t}+{ß}_{3}SIZE_{i,t}+{ß}_{4}LEV_{i,t}+{ß}_{5}GDP_{i,t}+\varepsilon_{i,t}$$ (9)

where i is the firm; t is the year; ß represents the presumed parameter; ε stands for the error term.

## 4. Results and discussion

### 4.1 Descriptive statistics

Our analysis starts with the descriptive statistics. Table 2 shows the descriptive statistics of variables in this study. During the observed period, the mean value of ROA is 0.036, which suggests that the profitability of Chinese agri-food companies is relatively low. This is in line with Xu and Jin [18]. Regarding WC policy variables, CATA and CLTA have mean values of 0.439 and 0.401, respectively. Adam et al. [69] argued that firms adopt conservative WC policies if CATA is higher than 50% and CLTA is less than 50%. It can be inferred that agri-food companies might face the constraints in obtaining short-term financing to operate. The mean CCC (86.544) suggests that agri-food companies take 86.5 days, on average, from purchasing raw materials to collecting sales proceeds. The mean value of NWC is 0.038, implying that such companies are operating efficiently and have ability to cope with their financial obligation. The mean value of SIZE is 22.241. LEV has a mean value of 2.909, which means that sampled companies tend to experience financial problems in meeting their debt payment obligations.

**Table 2:** Descriptive statistics.

Variable	N	Mean	Maximum	Minimum	SD	Skewness	Kurtosis
ROA	128	0.036	0.227	-0.369	0.084	-0.608	4.547
CATA	128	0.439	0.722	0.048	0.151	-0.497	-0.322
CLTA	128	0.401	0.862	0.005	0.182	-0.009	-0.377
CCC	128	86.544	412.449	4.754	89.544	1.469	1.536
NWC	128	0.038	0.400	-0.400	0.159	-0.524	0.022
SIZE	128	22.241	25.612	20.197	1.248	0.400	-0.543
LEV	128	2.909	173.618	0.049	15.655	10.457	113.829
GDP	128	0.083	0.142	0.022	0.027	0.189	0.683

Table 3 shows the results of normality test. Based on the skewness and kurtosis and Table 3, all variables except CLTA do not have the normal data distribution (*p* < 0.05).

**Table 3:** Normality test.

Variable	Statistic	df	Sig.
ROA	0.889	128	0.000
CATA	0.972	128	0.009
CLTA	0.990	128	0.470
CCC	0.808	128	0.000
NWC	0.973	128	0.012
SIZE	0.969	128	0.005
LEV	0.125	128	0.000
GDP	0.918	128	0.000

**Fig 1:**
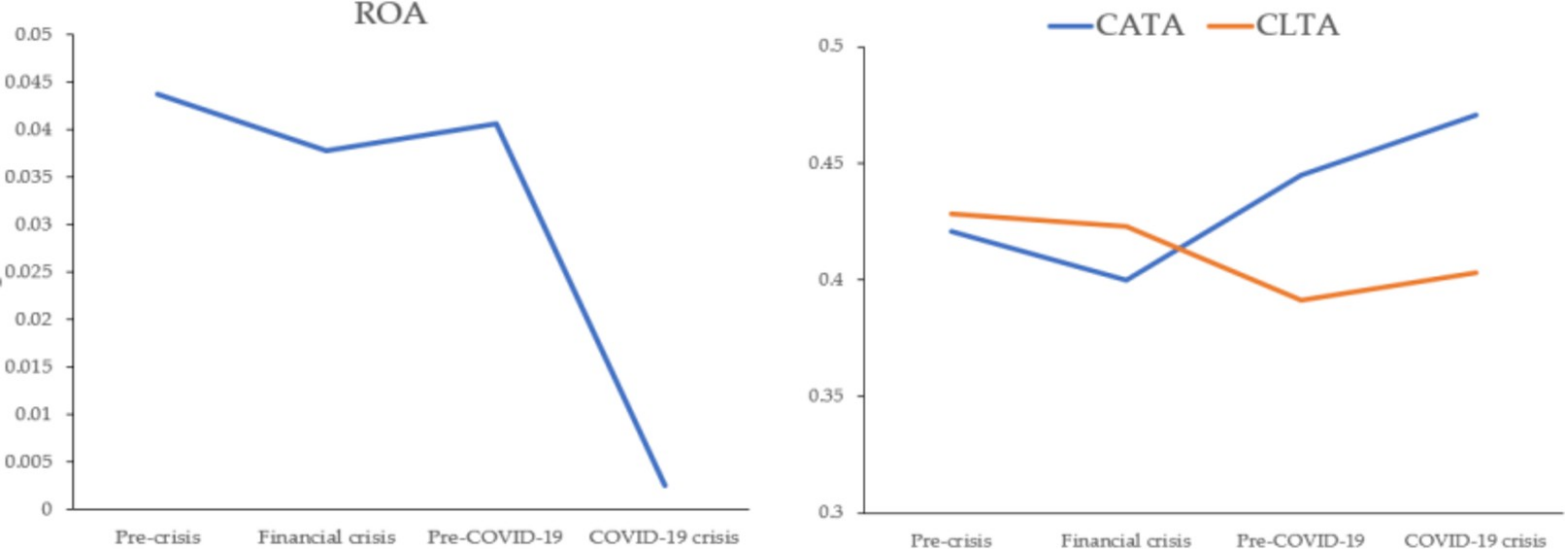
Changes in ROA, CATA, and CLTA during 2006–2021.

**Fig 2:**
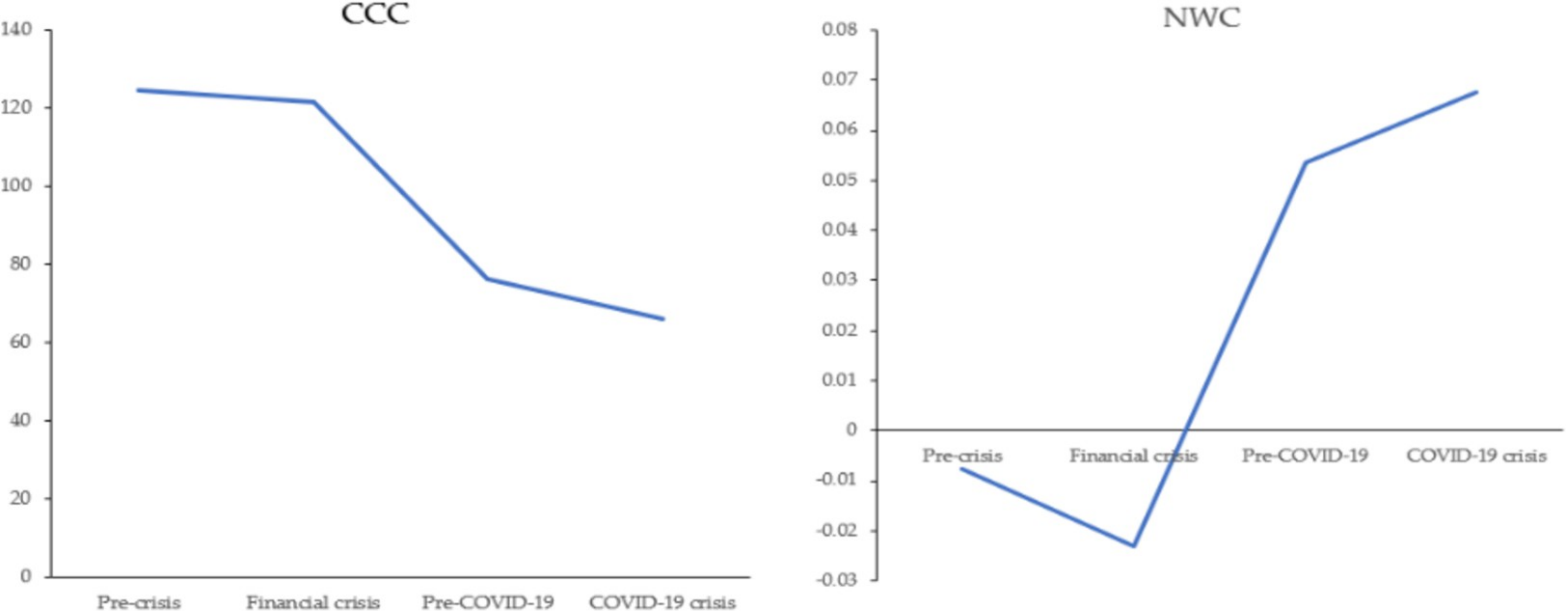
Changes in CCC and NWC during 2006–2021.

Table 4 compares WC practices and financial performance before the 2008 financial crisis, during the 2008 financial crisis, before COVID-19, and during COVID-19 using Kruskal-Wallis non-parametric test. The last column of Table 4 reveals that there is no significant change in the mean ranks among the four time periods. Figs 1 and 2 show the changes in the dependent and independent variables during the observed periods. For ROA, there was a decline in both recession periods, but this decline was more pronounced in the era of COVID-19. It can be inferred that the impact of COVID-19 on financial performance is stronger than the 2008 financial crisis. This is inconsistent with Ahmad et al. [5] who found that firms’ ROA in three Asian developing countries slightly increased in the 2008 financial crisis and highly decreased during COVID-19 crisis. During the 2008 financial crisis, CATA and CLTA decreased compared to the value pre-crisis, while they increased compared to the values before COVID-19. From pre-crisis to the COVID-19 crisis, such companies increased the investment in current assets. CCC has a declining trend during the four periods. NWC shows the decline in 2008 financial crisis, and the increase in the COVID-19 period, which implies that such companies have begun to realize the importance of net WCM during crisis. In 2008 financial crisis, firms seem to reduce WC to asset ratio [14].

**Table 4:** Kruskal-Wallis non-parametric test.

Variable	Pre-crisis (2006–2007)	Financial crisis (2008–2009)	Pre-COVID-19 (2010–2019)	COVID-19 crisis (2020–2021)	K-Wallis Stat. (*p* value)
ROA	0.0437	0.0378	0.0406	0.0025	2.464 (0.482)
CATA	0.4206	0.3998	0.4448	0.4705	3.889 (0.274)
CLTA	0.4282	0.4230	0.3913	0.4030	0.890 (0.828)
CCC	124.5656	121.6591	76.3783	66.2366	3.597 (0.308)
NWC	-0.0076	-0.0231	0.0535	0.0675	5.469 (0.141)

### 4.2 Correlation analysis

Table 5 shows the results of correlation analysis. ROA is positively related to NWC, and negatively related to CLTA and CCC. However, ROA is not related to CATA. SIZE has a positive relation with ROA, while LEV has a negative relation. In addition, all values of variance inflation factor (VIF) in Table 6 are calculated, and they are found to be less than 2, which suggests that multi-collinearity is not important.

**Table 5:** Correlation matrix.

Variable	ROA	CATA	CLTA	CCC	NWC	SIZE	LEV	GDP
ROA	1							
CATA	0.056	1						
CLTA	-0.300***	0.556***	1					
CCC	-0.228***	0.134*	0.157**	1				
NWC	0.396***	0.314***	-0.614***	-0.052	1			
SIZE	0.410***	0.044	0.058	-0.083	-0.025	1		
LEV	-0.315***	0.037	0.286***	-0.043	-0.291***	0.026	1	
GDP	0.006	-0.099	0.083	0.193**	-0.189**	-0.287***	-0.086	1

Notes

* *p* < 0.10

** *p* < 0.05

*** *p* < 0.01.

**Table 6:** VIF values.

Variable	VIF1	VIF2	VIF3
ROA	-	-	-
CATA	1.546	-	-
CLTA	1.700	-	-
CCC	-	1.040	-
NWC	-	-	1.159
SIZE	1.101	1.091	1.098
LEV	1.140	1.008	1.118
GDP	1.164	1.132	1.164

### 4.3 Results and discussion

Before regression analysis, we use the Chow test to determine whether the ordinary least squares (OLS) method or panel data method should be used. It shows that each research model has a *p*-value of less than 0.05, suggesting that the OLS method is accepted [70]. In addition, we use the White test to investigate heteroskedasticity problems. All models have *p*-values of greater than 0.05, indicating that there are no heteroskedasticity problems. Guided by Kraiczy et al. [71] and Delgado-García et al. [72], we use the Durbin-Wu-Hausman test for endogeneity of the independents, and find that OLS can provide unbiased estimates.

Table 7 presents the regression results of Models (1)–(3). Model (1) shows that CATA produces a significant and positive influence on firms’ ROA (ß = 0.180, t = 3.780), suggesting that higher current assets to total assets ratio leads to better financial performance. Therefore, H1 is fully supported. This can be achieved by increasing current asset levels with the adoption of conservative WC investment policy. This finding corroborates the findings of a great deal of previous work indicating that conservative WC investment policy improves financial performance [6, 50, 73, 74]. The coefficient of CLTA is negative and significant (ß = -0.217, t = -5.238), suggesting that firms’ financial performance can be enhanced by the utilization of less current liabilities to finance operating activities. Therefore, H2 is accepted. This finding agrees with studies by Ahmad et al. [5], Tahir and Anuar [33], Rasyid [34], and Basyith et al. [37] who found that conservative WC financing policy is beneficial to firms’ financial performance. Reyad et al. [73] pointed out that enterprises are more conservative in maintaining WCM during crisis in China.

**Table 7:** Regression results of Models (1)–(3).

Variable	Model (1)	Model (2)	Model (3)
Constant	-0.729*** (-6.279)	-0.638*** (-5.240)	-0.740*** (-6.415)
CATA	0.180*** (3.780)		
CLTA	-0.217*** (-5.238)		
CCC		-0.0002*** (-3.122)	
NWC			0.204*** (5.233)
SIZE	0.033*** (6.707)	0.030*** (5.766)	0.032** (6.675)
LEV	-0.001** (-2.552)	-0.002*** (-4.393)	-0.001*** (-2.707)
GDP	0.619*** (2.688)	0.464* (1.921)	0.621*** (2.697)
Adj. R^2^	0.395	0.316	0.396
F	17.598***	15.652***	21.816***
N	128	128	128

Notes

* *p* < 0.10

** *p* < 0.05

*** *p* < 0.01. *t*-values are in parentheses.

Model (2) shows a negative CCC-ROA relationship (ß = -0.0002, t = -3.122), which suggests that shortening the CCC can increase financial performance. Therefore, H3 is accepted. The shorter CCC means the shorter inventory conversion cycle and receivable collection cycle and the longer payable deferral cycle. It will result in the lesser blocked funds in WC and reduce the need to use external capital to finance operations. This is in agreement with Iotti and Bonazzi [75] who found that the duration of CCC negatively influences ROE in agri-food companies. However, this result is contrary to the studies by Liu and Xu [2], Respati et al. [3], Laghari and Chengang [48], Wassie [49], and Korent and Orsag [50]. During the lock-in period, Tarkom [68] concluded that US firms operate with higher level of CCC. Regarding NWC, it has a significant and positive impact on the ROA indicator (ß = 0.204, t = 5.233). Therefore, H4 is accepted. This suggests that agri-food companies with greater proportion of current assets outperform others. This parallels the findings of Senan et al. [51] and Asche et al. [76]. In addition, SIZE is positively associated with ROA, whereas LEV is negatively associated. GDP has a positive influence on ROA.

Table 8 shows the regression results of Models (4)–(9) that examine the impact of the 2008 financial crisis and the COVID-19 crisis on the WC-financial performance relationship. In Models (4)–(6), the coefficients of CRISIS are not significant at the 5% level. Akgün and Karataş [13] argued that the 2008 financial crisis is negatively related to ROA in EU-28 listed firms. However, the coefficients of COVID are significant and negative. This means that COVID-19 hits firms’ financial performance more seriously than the 2008 financial crisis, which accords with the results of Ahmad et al. [5]. However, Xu and Jin [18] observed that the COVID-19 outbreak does not significantly influence financial performance of agri-food companies.

**Table 8:** Regression results of Models (4)–(9).

Variable	Model (4)	Model (5)	Model (6)	Model (7)	Model (8)	Model (9)
Constant	-0.673*** (-5.384)	-0.667*** (-5.308)	-0.669*** (-5.372)	-0.614*** (-5.102)	-0.652*** (-5.333)	-0.614*** (-5.106)
CRISIS	-0.027 (-0.409)	0.043 (1.622)	0.017 (0.871)			
CRISIS×CATA	0.335** (2.229)					
CRISIS×CLTA	-0.228* (-1.812)					
CRISIS×CCC		-0.0003* (-1.776)				
CRISIS×NWC			0.266** (2.341)			
COVID				-0.112** (-2.498)	-0.080*** (-2.954)	-0.074*** (-3.525)
COVID×CATA				0.286*** (2.728)		
COVID×CLTA				-0.200** (-2.018)		
COVID×CCC					0.0003 (1.160)	
COVID×NWC						0.237** (2.595)
SIZE	0.031*** (5.832)	0.031*** (5.754)	0.031*** (5.821)	0.030*** (5.803)	0.031*** (6.038)	0.030*** (5.810)
LEV	-0.002*** (-4.185)	-0.002*** (-4.172)	-0.002*** (-4.190)	-0.002*** (-4.272)	-0.002*** (-4.376)	-0.002*** (-4.237)
GDP	0.321 (1.309)	0.316 (1.279)	0.319 (1.304)	0.036 (0.139)	0.056 (0.213)	0.032 (0.124)
Adj. R^2^	0.286	0.276	0.289	0.338	0.310	0.339
F	9.489***	10.683***	11.335***	11.829***	12.417***	14.026***
N	128	128	128	128	128	128

Notes

* *p* < 0.10

** *p* < 0.05

*** *p* < 0.01. *t*-values are in parentheses.

All coefficients of the interaction in Models (4)–(6) are higher than those in Models (7)–(9), indicating that the influence of these two crises on the WC-financial performance relationship is different. H5, H6, H7, and H8 are preliminarily supported. Tsuruta [11] found that the negative CCC-firm performance relationship became more intensified in the 2008 crisis, but this relationship existed for a short time. This echoes the findings of Song et al. [77] who argued that economic crisis caused by COVID-19 differs from past financial ones.

As of control variables, during the two crisis periods, SIZE positively affects financial performance, while LEV exhibits a negative impact in Models (4)–(9). Overall, we find that the relationship between WC and financial performance changes because of economic conditions.

We future investigate the impact of WC on financial performance during two periods (2008–2009 and 2020–2021), respectively. The regression results of two crisis periods are exhibited in Table 9. During 2008–2009, CATA and CLTA have no significant impact on the ROA indicator, while CATA has a positive impact and CLTA has a negative impact during COVID-19. Therefore, H5 and H6 are supported. As Safitri et al. [64] claimed, WC investment and financing strategies improve ROA before and during COVID-19 in Indonesia. NWC positively influences ROA only in the era of COVID-19, which leads to the acceptance of H8. However, CCC decreases ROA in the 2008 financial crisis, while it has no impact during 2020–2021. Therefore, H7 is not supported. The findings support evidence from Song et al. [77]. Agri-food companies implemented effective WCM by shortening CCC and improving NWC during COVID-19, which leads to the insignificant of CCC on ROA. It was reported that CCC of European listed firms dropped by 8.7 days in 2020 [78]. Improving the CCC reduces WC requirements, which leads to increased profitability. Businesses and individuals have a higher demand for food and household products [79]. This also forces agri-food companies to improve the efficiency of WCM to ensure that food supply chain continues to function.

**Table 9:** Regression results of two crisis periods.

Variable	Financial crisis (2008–2009)			COVID-19 crisis (2020–2021)		
	Model (1)	Model (2)	Model (3)	Model (1)	Model (2)	Model (3)
Constant	0.158 (0.104)	0.677 (0.521)	-0.493 (-0.328)	-0.417 (-1.229)	-0.721 (-1.699)	-0.471 (-1.460)
CATA	0.254 (1.553)			0.553** (3.018)		
CLTA	0.095 (0.291)			-0.649** (-2.773)		
CCC		-0.0003** (-2.258)			0.0001 (0.271)	
NWC			0.288 (1.723)			0.545** (3.048)
SIZE	0.044* (2.101)	0.038* (1.920)	0.042* (1.944)	0.018 (1.241)	0.035* (1.893)	0.020 (1.363)
LEV	-0.100 (-1.055)	-0.064* (-2.032)	0.008 (0.157)	0.015* (2.103)	-0.001 (-0.212)	0.013* (2.091)
GDP	-11.804 (-0.806)	-14.426 (-1.167)	-4.153 (-0.297)	-0.886 (-1.157)	-1.461 (-1.437)	-0.948 (-1.275)
Adj. R^2^	0.338	0.383	0.288	0.504	0.131	0.526
F	2.533**	3.323**	2.520**	4.043**	1.565*	5.157**

Notes

* *p* < 0.10

** *p* < 0.05. *t*-values are in parentheses.

Hamshari et al. [80] argued that COVID-19 leads to the decline in WC performance, and a conservative WCM method is appropriate for most companies during the COVID-19 period. However, Habib and Kayani [81] concluded that COVID-19 does not significantly affects the efficiency of WCM. A Romanian study showed that NWC increases in 2020 compared to 2019 and it positively impacts corporate performance [82]. Chen and Xu [83], taking Chinese manufacturing companies as the sample, also found a positive relationship between NWC and ROA. Overall, these results also suggest that the COVID-19 had detrimental consequences for firm governance and performance.

### 4.4 Robustness check

ROE is used to replace ROA as the dependent variable, and all models are re-estimated. The results are similar to previous findings. In addition, all independent variables are lagged for one year, and all models are re-estimated. The results are also found to be similar to previous findings.

## 5. Conclusions

This study aims to explore what kind of WCM policies is adopted by Chinese agri-food companies during 2006–2021. In addition, this study examines whether the impact of WC on financial performance is different during the 2008 financial crisis and COVID-19 crisis. The main results are listed as follows. Firstly, the WC-financial performance relationship is more affected during COVID-19 than in the 2008 financial crisis. In addition, current assets to total assets ratio and NWC positively influence financial performance measured through ROA, while current liabilities to total assets ratio and CCC have a positive and significant. Secondly, there is a difference in the impact of WC on financial performance during COVID-19 as compared to the 2008 financial crisis.

The theoretical contributions can be discussed as follows. First, this study first empirically explores the relationship between WCM and firms’ financial performance in China’s agri-food sector during the 2008 financial crisis and the COVID-19 crisis, which enriches the current WCM literature. Second, it might provide insights for agri-food companies to help them manage WC during the crisis. Also, it could become a base for future researchers who attempt to explore the WCM during crisis times in other emerging markets.

The practical implications are put forward as follows. First of all, agri-food companies should pay attention to managing current assets and current liabilities during crisis times. Managers should hold sufficient current assets during the crisis in case that current liabilities are due. Second, agri-food companies need to pay attention to the appropriate levels in the components of WCM. In crisis, they should focus on the availability of cash, extend payment terms, write off early payments, reduce unnecessary inventory, and intensify collection. Additionally, corporate management need to promptly adjust their WCM strategies according to external environment. Finally, the government should issue some favorable policies to alleviate corporate financial pressure and provide funding supports to help companies recover from the tough times.

The limitations of this study are listed in two aspects. First, in this study, only agri-food sector is used as the sample, and future research can extend the sample to other industries. In addition, more measures of financial performance can be used in future research. Second, future research can use survey data to have a deep understanding on how managers behave in managing WC during crisis times.

## Supporting information

S1 Data (XLS)
